# Final 2012 Reports of Nationally Notifiable Infectious Diseases

**Published:** 2013-08-23

The tables listed in this report on pages 970–982 summarize finalized data, as of June 30, 2013, from the National Notifiable Diseases Surveillance System (NNDSS) for 2012. These data will be published in more detail in the *Summary of Notifiable Diseases — United States, 2012* (1). Because no cases were reported in the United States during 2012, the following diseases do not appear in these early release tables: anthrax; eastern equine encephalitis virus disease, nonneuroinvasive; poliomyelitis, paralytic; poliovirus infection, nonparalytic; Powassan virus nonneuroinvasive disease; severe acute respiratory syndrome–associated coronavirus disease (SARS-CoV); smallpox; western equine encephalitis virus disease, neuroinvasive and nonneuroinvasive; yellow fever; and viral hemorrhagic fevers.

Policies for reporting NNDSS data to CDC can vary by disease or reporting jurisdiction depending on case status classification (i.e., confirmed, probable, or suspected). The publication criteria used for the 2012 finalized tables are listed in the “Print Criteria” column of the NNDSS event code list, available at http://wwwn.cdc.gov/nndss/document/nndss_event_code_list_2012.pdf. In addition, only cases from reporting jurisdictions where the nationally notifiable disease is reportable are published. The NNDSS website is updated annually to include the latest national surveillance case definitions approved by the Council of State and Territorial Epidemiologists for classifying and enumerating cases of nationally notifiable infectious diseases.

Population estimates are from the National Center for Health Statistics postcensal estimates of the resident population of the United States for July 1, 2011–July 1, 2012, by year, county, single-year of age (0 to ≥85 years), bridged-race, (white, black or African American, American Indian or Alaska Native, Asian or Pacific Islander), Hispanic origin (not Hispanic or Latino, Hispanic or Latino), and sex (vintage 2011), prepared under a collaborative arrangement with the U.S. Census Bureau. Population estimates for states are available at http://www.cdc.gov/nchs/nvss/bridged_race/data_documentation.htm#vintage2011 as of June 13, 2013. Population estimates for territories are 2012 estimates from the U.S. Census Bureau (2).

## Figures and Tables

**TABLE 2:** Reported cases of notifiable diseases,* by geographic division and area — United States, 2012

Area	Total Resident Population (in thousands)	Arboviral diseases†							

		California serogroup viruses		Eastern equine encephalitis virus	Powassan virus	St. Louis encephalitis virus		West Nile virus	
				
		Neuro-invasive	Nonneuro-invasive	Neuro-invasive	Neuro-invasive	Neuro-invasive	Nonneuro-invasive	Neuro-invasive	Nonneuro-invasive
**United States**	311,589	73	8	15	7	1	2	2,872	2,801
**New England**	14,519	—	—	9	—	—	—	42	21
Connecticut	3,587	—	—	—	—	—	—	12	9
Maine	1,329	—	—	—	—	—	—	1	—
Massachusetts	6,607	—	—	7	—	—	—	25	8
New Hampshire	1,318	—	—	—	—	—	—	1	—
Rhode Island	1,051	—	—	—	—	—	—	2	2
Vermont	627	—	—	2	—	—	—	1	2
**Mid. Atlantic**	41,081	1	—	—	1	—	—	116	99
New Jersey	8,835	—	—	—	—	—	—	22	26
New York (Upstate)	11,232	1	—	—	1	—	—	35	31
New York City	8,270	—	—	—	—	—	—	26	15
Pennsylvania	12,744	—	—	—	—	—	—	33	27
**E.N. Central**	46,504	16	3	—	2	—	—	494	253
Illinois	12,860	—	—	—	—	—	—	187	103
Indiana	6,516	2	1	—	—	—	—	46	31
Michigan	9,877	—	—	—	—	—	—	141	61
Ohio	11,541	12	1	—	—	—	—	76	45
Wisconsin	5,710	2	1	—	2	—	—	44	13
**W.N. Central**	20,641	4	—	—	4	—	—	225	437
Iowa	3,064	—	—	—	—	—	—	11	20
Kansas	2,870	—	—	—	—	—	—	20	36
Minnesota	5,347	4	—	—	4	—	—	34	36
Missouri	6,009	—	—	—	—	—	—	17	3
Nebraska	1,842	—	—	—	—	—	—	42	151
North Dakota	685	—	—	—	—	—	—	39	50
South Dakota	824	—	—	—	—	—	—	62	141
**S. Atlantic**	60,544	39	5	6	—	—	—	185	129
Delaware	908	—	—	—	—	—	—	2	7
District of Columbia	619	—	—	—	—	—	—	8	2
Florida	19,082	—	—	2	—	—	—	52	21
Georgia	9,812	—	—	1	—	—	—	46	53
Maryland	5,840	—	—	—	—	—	—	25	22
North Carolina	9,651	26	—	2	—	—	—	7	—
South Carolina	4,673	2	—	—	—	—	—	20	9
Virginia	8,104	2	—	1	—	—	—	20	10
West Virginia	1,855	9	5	—	—	—	—	5	5
**E.S. Central**	18,548	10	—	—	—	—	—	173	192
Alabama	4,804	—	—	—	—	—	—	38	24
Kentucky	4,367	—	—	—	—	—	—	13	10
Mississippi	2,977	1	—	—	—	—	—	103	144
Tennessee	6,400	9	—	—	—	—	—	19	14
**W.S. Central**	36,930	3	—	—	—	1	2	1,146	1,312
Arkansas	2,939	—	—	—	—	—	—	44	20
Louisiana	4,575	—	—	—	—	—	—	155	180
Oklahoma	3,784	—	—	—	—	—	—	103	88
Texas	25,632	3	—	—	—	1	2	844	1,024
**Mountain**	22,345	—	—	—	—	—	—	190	165
Arizona	6,467	—	—	—	—	—	—	87	46
Colorado	5,116	—	—	—	—	—	—	62	69
Idaho	1,584	—	—	—	—	—	—	5	12
Montana	998	—	—	—	—	—	—	1	5
Nevada	2,720	—	—	—	—	—	—	5	4
New Mexico	2,079	—	—	—	—	—	—	24	23
Utah	2,814	—	—	—	—	—	—	3	2
Wyoming	567	—	—	—	—	—	—	3	4
**Pacific**	50,477	—	—	—	—	—	—	301	193
Alaska	724	—	—	—	—	—	—	—	—
California	37,684	—	—	—	—	—	—	297	182
Hawaii	1,378	—	—	—	—	—	—	—	—
Oregon	3,868	—	—	—	—	—	—	—	11
Washington	6,823	—	—	—	—	—	—	4	—

**Territories**									
American Samoa	55	—	—	—	—	—	—	—	—
C.N.M.I.	52	—	—	—	—	—	—	—	—
Guam	160	—	—	—	—	—	—	—	—
Puerto Rico	3,707	—	—	—	—	—	—	1	—
U.S. Virgin Islands	106	—	—	—	—	—	—	—	—

	Babesiosis			Botulism					
				
Area	Total	Confirmed	Probable	Total	Foodborne	Infant	Other†	Brucellosis	Chancroid§
**United States**	940	717	223	168	27	123	18	114	15
**New England**	471	418	53	2	—	2	—	—	1
Connecticut	123	106	17	1	—	1	—	—	—
Maine	10	8	2	—	—	—	—	—	—
Massachusetts	261	237	24	1	—	1	—	—	1
New Hampshire	19	18	1	—	—	—	—	—	—
Rhode Island	56	47	9	—	—	—	—	—	—
Vermont	2	2	—	—	—	—	—	—	—
**Mid. Atlantic**	346	224	122	37	2	34	1	4	—
New Jersey	92	75	17	7	1	6	—	—	—
New York (Upstate)	226	130	96	5	—	4	1	—	—
New York City	28	19	9	4	1	3	—	3	—
Pennsylvania	N	N	N	21	—	21	—	1	—
**E.N. Central**	72	55	17	11	4	7	—	10	3
Illinois	2	1	1	1	—	1	—	5	—
Indiana	1	—	1	—	—	—	—	3	1
Michigan	—	—	—	4	2	2	—	1	2
Ohio	N	N	N	6	2	4	—	—	—
Wisconsin	69	54	15	—	—	—	—	1	—
**W.N. Central**	41	14	27	2	—	1	1	2	—
Iowa	N	N	N	—	—	—	—	—	—
Kansas	N	N	N	1	—	—	1	1	—
Minnesota	40	13	27	—	—	—	—	—	—
Missouri	N	N	N	1	—	1	—	1	—
Nebraska	1	1	—	—	—	—	—	—	—
North Dakota	—	—	—	—	—	—	—	—	—
South Dakota	N	N	N	—	—	—	—	—	—
**S. Atlantic**	6	2	4	10	1	8	1	28	3
Delaware	—	—	—	1	1	—	—	—	—
District of Columbia	3	1	2	1	—	1	—	—	—
Florida	N	N	N	1	—	1	—	17	—
Georgia	N	N	N	1	—	1	—	4	—
Maryland	3	1	2	2	—	2	—	1	1
North Carolina	N	N	N	1	—	1	—	5	1
South Carolina	N	N	N	1	—	—	1	—	—
Virginia	N	N	N	2	—	2	—	—	1
West Virginia	N	N	N	—	—	—	—	1	—
**E.S. Central**	—	—	—	7	—	7	—	2	1
Alabama	—	—	—	—	—	—	—	—	1
Kentucky	N	N	N	4	—	4	—	1	—
Mississippi	N	N	N	2	—	2	—	—	—
Tennessee	—	—	—	1	—	1	—	1	—
**W.S. Central**	—	—	—	4	—	3	1	22	—
Arkansas	N	N	N	1	—	1	—	2	—
Louisiana	N	N	N	—	—	—	—	2	—
Oklahoma	N	N	N	1	—	1	—	—	—
Texas	N	N	N	2	—	1	1	18	—
**Mountain**	—	—	—	30	12	18	—	9	—
Arizona	N	N	N	14	12	2	—	5	—
Colorado	N	N	N	1	—	1	—	2	—
Idaho	N	N	N	2	—	2	—	—	—
Montana	—	—	—	—	—	—	—	—	—
Nevada	N	N	N	—	—	—	—	—	—
New Mexico	N	N	N	2	—	2	—	—	—
Utah	N	N	N	9	—	9	—	2	—
Wyoming	—	—	—	2	—	2	—	—	—
**Pacific**	4	4	—	65	8	43	14	37	7
Alaska	N	N	N	3	3	—	—	1	—
California	4	4	—	52	3	36	13	34	7
Hawaii	N	N	N	—	—	—	—	2	—
Oregon	—	—	—	4	1	3	—	—	—
Washington	—	—	—	6	1	4	1	—	—

**Territories**									
American Samoa	U	U	U	—	—	—	—	—	—
C.N.M.I.	—	—	—	—	—	—	—	—	—
Guam	—	—	—	—	—	—	—	—	—
Puerto Rico	N	N	N	N	N	N	N	—	—
U.S. Virgin Islands	N	N	N	—	—	—	—	—	—

Area	*Chlamydia trachomatis* infection†	Cholera	Coccidioidomycosis	Cryptosporidiosis			Cyclosporiasis

				Total	Confirmed	Probable	
**United States**	1,422,976	17	17,802	7,956	5,098	2,718	123
**New England**	49,137	1	3	391	333	58	7
Connecticut	13,065	—	N	41	41	—	6
Maine	3,413	—	N	58	32	26	N
Massachusetts	23,550	1	—	155	155	—	1
New Hampshire	3,072	—	2	54	22	32	—
Rhode Island	4,313	—	1	16	16	—	—
Vermont	1,724	—	N	67	67	—	N
**Mid. Atlantic**	182,810	6	4	809	669	140	28
New Jersey	27,271	—	N	42	41	1	7
New York (Upstate)	38,227	—	N	229	220	9	5
New York City	62,319	4	N	124	121	3	16
Pennsylvania	54,993	2	4	414	287	127	N
**E.N. Central**	221,639	—	51	1,863	1,154	709	2
Illinois	67,701	—	N	173	73	100	2
Indiana	29,505	—	N	164	127	37	—
Michigan	47,566	—	27	351	285	66	—
Ohio	53,141	—	22	569	64	505	—
Wisconsin	23,726	—	2	606	605	1	—
**W.N. Central**	81,983	—	151	1,349	626	723	2
Iowa	11,377	—	N	328	78	250	—
Kansas	11,135	—	N	122	65	57	—
Minnesota	18,056	—	119	347	213	134	—
Missouri	27,835	—	17	239	109	130	2
Nebraska	6,748	—	1	165	127	38	—
North Dakota	2,908	—	14	35	3	32	N
South Dakota	3,924	—	N	113	31	82	—
**S. Atlantic**	285,340	9	9	1,142	686	456	34
Delaware	4,438	—	1	15	7	8	—
District of Columbia	6,808	—	1	N	N	N	N
Florida	77,644	7	N	470	243	227	25
Georgia	52,418	—	N	257	257	—	2
Maryland	26,534	2	7	86	30	56	4
North Carolina	50,596	—	N	86	28	58	2
South Carolina	27,149	—	N	72	36	36	—
Virginia	34,963	—	N	144	81	63	1
West Virginia	4,790	—	N	12	4	8	—
**E.S. Central**	103,473	—	—	284	148	136	2
Alabama	30,621	—	N	109	19	90	N
Kentucky	17,273	—	N	63	50	13	N
Mississippi	23,054	—	N	40	40	—	N
Tennessee	32,525	—	N	72	39	33	2
**W.S. Central**	187,843	1	4	596	482	114	45
Arkansas	16,611	—	N	42	41	1	—
Louisiana	27,353	—	4	155	155	—	1
Oklahoma	16,843	—	N	97	14	83	—
Texas	127,036	1	N	302	272	30	44
**Mountain**	93,204	—	13,140	830	676	154	1
Arizona	30,444	—	12,920	47	35	12	—
Colorado	21,631	—	N	102	63	39	1
Idaho	4,550	—	N	267	182	85	N
Montana	3,827	—	3	69	69	—	N
Nevada	11,137	—	118	15	9	6	N
New Mexico	11,898	—	37	94	91	3	—
Utah	7,615	—	56	202	196	6	—
Wyoming	2,102	—	6	34	31	3	—
**Pacific**	217,547	—	4,440	692	324	228	2
Alaska	5,462	—	N	7	7	—	N
California	167,695	—	4,431	365	216	9	1
Hawaii	6,340	—	N	5	5	—	—
Oregon	13,454	—	2	214	16	198	1
Washington	24,596	—	7	101	80	21	—

**Territories**	—	—	N	N	N	N	N
American Samoa	—	—	—	—	—	—	—
C.N.M.I.	1,031	—	—	—	—	—	—
Guam	6,227	—	N	N	N	N	N
Puerto Rico	802	—	—	—	—	—	—
U.S. Virgin Islands	802	—	—	—	—	—	—

	Dengue Virus Infection†			Ehrlichiosis/Anaplasmosis			
			
Area	Dengue Fever	Dengue Hemorrhagic Fever	Diphtheria	*Anaplasma phagocytophilum*	*Ehrlichia chaffeensis*	*Ehrlichia ewingii*	Undetermined
**United States**	544	3	1	2,389	1,128	17	191
**New England**	17	—	—	659	52	—	—
Connecticut	16	—	—	142	—	—	—
Maine	—	—	—	52	3	—	—
Massachusetts	—	—	—	318	25	—	—
New Hampshire	—	—	—	52	3	—	—
Rhode Island	—	—	—	86	21	—	—
Vermont	1	—	—	9	—	—	—
**Mid. Atlantic**	132	—	1	482	123	—	31
New Jersey	—	—	—	139	58	—	1
New York (Upstate)	16	—	1	315	48	—	13
New York City	95	—	—	20	11	—	—
Pennsylvania	21	—	—	8	6	—	17
**E.N. Central**	55	1	—	604	61	1	102
Illinois	20	1	—	12	36	1	1
Indiana	9	—	—	—	—	—	35
Michigan	9	—	—	6	2	—	—
Ohio	6	—	—	1	3	—	1
Wisconsin	11	—	—	585	20	—	65
**W.N. Central**	19	1	—	538	236	11	27
Iowa	2	—	—	N	N	N	N
Kansas	1	—	—	7	41	1	—
Minnesota	9	—	—	503	9	—	17
Missouri	5	1	—	23	186	10	9
Nebraska	—	—	—	2	—	—	—
North Dakota	—	—	—	3	—	—	—
South Dakota	2	—	—	—	—	—	1
**S. Atlantic**	185	—	—	56	334	2	11
Delaware	—	—	—	1	16	1	—
District of Columbia	—	—	—	N	N	N	N
Florida	139	—	—	5	23	—	—
Georgia	11	—	—	5	24	—	2
Maryland	9	—	—	5	37	—	—
North Carolina	7	—	—	21	109	—	2
South Carolina	2	—	—	—	2	—	—
Virginia	17	—	—	18	123	1	6
West Virginia	—	—	—	1	—	—	1
**E.S. Central**	12	—	—	26	102	3	11
Alabama	4	—	—	11	10	—	5
Kentucky	1	—	—	1	29	—	—
Mississippi	1	—	—	1	2	—	—
Tennessee	6	—	—	13	61	3	6
**W.S. Central**	23	—	—	24	220	—	1
Arkansas	—	—	—	8	85	—	—
Louisiana	6	—	—	—	1	—	1
Oklahoma	1	—	—	15	130	—	—
Texas	16	—	—	1	4	—	—
**Mountain**	13	—	—	—	—	—	2
Arizona	8	—	—	—	—	—	1
Colorado	—	—	—	N	N	N	N
Idaho	1	—	—	N	N	N	N
Montana	2	—	—	N	N	N	N
Nevada	2	—	—	—	—	—	—
New Mexico	—	—	—	N	N	N	N
Utah	—	—	—	—	—	—	1
Wyoming	—	—	—	—	—	—	—
**Pacific**	88	1	—	—	—	—	6
Alaska	1	—	—	N	N	N	N
California	64	—	—	—	—	—	6
Hawaii	8	—	—	N	N	N	N
Oregon	—	—	—	—	—	—	—
Washington	15	1	—	—	—	—	—

**Territories**							
American Samoa	—	—	—	N	N	N	N
C.N.M.I.	—	—	—	—	—	—	—
Guam	—	—	—	N	N	N	N
Puerto Rico	5,907	118	—	N	N	N	N
U.S. Virgin Islands	141	1	—	—	—	—	—

Area	Giardiasis	Gonorrhea†	*Haemophilus influenza*, invasive disease				Hansen disease (leprosy)

			All ages, serotypes	Age <5 years			

				Serotype b	Nonserotype b	Unknown serotype	
**United States**	15,178	334,826	3,418	30	205	210	82
**New England**	1,436	5,970	235	2	13	7	3
Connecticut	223	2,133	61	—	—	3	1
Maine	169	456	23	—	2	—	N
Massachusetts	698	2,628	111	2	10	—	1
New Hampshire	105	147	12	—	—	4	1
Rhode Island	58	507	19	—	—	—	—
Vermont	183	99	9	—	1	—	N
**Mid. Atlantic**	2,928	45,447	674	8	29	24	5
New Jersey	423	7,486	124	—	—	11	—
New York (Upstate)	975	7,884	201	4	9	3	N
New York City	872	14,687	123	—	—	7	4
Pennsylvania	658	15,390	226	4	20	3	1
**E.N. Central**	2,203	59,268	570	6	40	41	2
Illinois	347	18,149	159	1	11	13	—
Indiana	227	7,338	104	2	13	1	—
Michigan	547	12,584	82	—	—	16	2
Ohio	578	16,493	158	3	16	—	—
Wisconsin	504	4,704	67	—	—	11	—
**W.N. Central**	1,726	17,676	245	2	7	23	4
Iowa	251	2,006	—	—	—	—	—
Kansas	133	2,228	32	—	1	3	—
Minnesota	610	3,082	85	2	6	6	1
Missouri	330	7,889	82	—	—	8	3
Nebraska	194	1,429	31	—	—	3	—
North Dakota	64	335	15	—	—	3	N
South Dakota	144	707	—	—	—	—	—
**S. Atlantic**	2,438	73,447	818	3	30	55	12
Delaware	24	899	8	—	1	—	—
District of Columbia	77	2,402	3	—	—	1	—
Florida	1,095	19,462	229	—	—	24	10
Georgia	544	15,326	186	—	8	16	1
Maryland	239	5,686	87	1	7	—	—
North Carolina	N	14,318	99	—	—	11	1
South Carolina	128	7,638	72	1	4	3	—
Virginia	272	6,885	101	—	8	—	—
West Virginia	59	831	33	1	2	—	N
**E.S. Central**	178	29,526	220	1	16	3	2
Alabama	178	9,270	55	1	2	1	1
Kentucky	N	4,283	36	—	1	—	—
Mississippi	N	6,875	26	—	6	—	1
Tennessee	N	9,098	103	—	7	2	—
**W.S. Central**	332	50,094	207	—	16	11	14
Arkansas	108	4,307	30	—	1	3	1
Louisiana	224	8,873	57	—	—	8	3
Oklahoma	N	4,441	117	—	15	—	N
Texas	N	32,473	3	—	N	N	10
**Mountain**	1,199	13,576	307	5	47	7	3
Arizona	113	5,809	120	2	23	1	—
Colorado	356	2,822	58	—	4	—	—
Idaho	153	167	18	—	3	1	—
Montana	67	108	6	—	1	—	—
Nevada	91	2,264	21	—	1	1	2
New Mexico	95	1,883	46	1	8	1	—
Utah	287	479	33	2	6	3	1
Wyoming	37	44	5	—	1	—	—
**Pacific**	2,738	39,822	142	3	7	39	37
Alaska	96	726	15	—	—	5	—
California	1,715	33,579	32	—	—	30	13
Hawaii	34	815	22	—	—	4	24
Oregon	381	1,464	69	2	4	—	N
Washington	512	3,238	4	1	3	—	N

**Territories**							
American Samoa	—	—	—	—	—	—	—
C.N.M.I.	—	—	—	—	—	—	—
Guam	2	92	—	—	—	—	10
Puerto Rico	24	345	—	—	—	—	—
U.S. Virgin Islands	—	136	N	N	N	N	—

Area	Hantavirus Pulmonary Syndrome	Hemolytic uremic syndrome, postdiarrheal	Hepatitis, viral, acute			Hepatitis B Perinatal infection	HIV diagnoses†

			A	B	C		
**United States**	30	274	1,562	2,895	1,782	40	35,361
**New England**	—	10	83	105	85	—	935
Connecticut	N	2	23	15	34	—	277
Maine	—	2	9	9	8	—	38
Massachusetts	—	5	40	75	37	—	510
New Hampshire	—	—	6	4	N	—	44
Rhode Island	—	—	3	U	U	—	62
Vermont	—	1	2	2	6	—	4
**Mid. Atlantic**	2	16	233	246	230	12	5,616
New Jersey	—	3	60	70	71	2	990
New York (Upstate)	1	12	63	50	83	3	1,327
New York City	—	1	48	63	10	4	2,026
Pennsylvania	1	N	62	63	66	3	1,273
**E.N. Central**	1	42	235	457	245	4	3,771
Illinois	1	7	67	86	26	1	1,388
Indiana	—	12	11	90	110	—	472
Michigan	—	5	100	81	76	2	654
Ohio	—	10	36	178	7	1	1,013
Wisconsin	—	8	21	22	26	—	244
**W.N. Central**	2	52	89	99	62	2	1,161
Iowa	1	10	7	13	3	—	116
Kansas	—	7	15	9	16	—	147
Minnesota	—	13	29	17	32	1	308
Missouri	—	18	20	48	4	1	496
Nebraska	—	1	16	10	3	—	58
North Dakota	—	3	2	—	—	—	9
South Dakota	1	—	—	2	4	—	27
**S. Atlantic**	1	26	267	754	423	5	10,327
Delaware	—	—	9	11	—	1	136
District of Columbia	N	N	—	—	—	—	509
Florida	—	1	87	247	107	1	4,629
Georgia	—	7	46	109	82	1	1,236
Maryland	—	4	28	52	39	—	1,016
North Carolina	—	7	34	73	63	—	1,145
South Carolina	—	4	6	37	1	—	716
Virginia	—	3	49	84	76	2	871
West Virginia	1	—	8	141	55	—	69
**E.S. Central**	—	26	78	577	331	1	2,120
Alabama	N	7	19	79	24	—	545
Kentucky	—	N	25	180	178	1	312
Mississippi	N	1	11	78	U	N	441
Tennessee	—	18	23	240	129	—	822
**W.S. Central**	—	26	161	367	140	6	5,118
Arkansas	—	3	8	74	5	1	125
Louisiana	—	1	7	44	11	—	1,156
Oklahoma	—	9	12	79	80	1	253
Texas	—	13	134	170	44	4	3,584
**Mountain**	11	17	163	89	112	—	1,504
Arizona	1	2	93	14	U	—	590
Colorado	3	6	28	24	42	—	362
Idaho	—	3	11	5	11	—	24
Montana	3	1	6	2	9	—	20
Nevada	—	—	10	28	12	—	326
New Mexico	1	—	10	3	21	—	109
Utah	2	5	4	13	17	—	65
Wyoming	1	—	1	—	—	—	8
**Pacific**	13	59	253	201	154	10	4,809
Alaska	N	N	1	1	—	—	26
California	9	44	209	136	63	7	4,037
Hawaii	—	—	5	5	—	1	43
Oregon	2	15	9	25	37	—	205
Washington	2	—	29	34	54	2	498

**Territories**							
American Samoa	N	N	—	—	—	—	—
C.N.M.I.	—	—	—	—	—	—	—
Guam	N	—	—	—	—	—	2
Puerto Rico	—	N	6	32	N	—	507
U.S. Virgin Islands	—	N	—	—	—	—	8

Area	Influenza-associated pediatric mortality†	Invasive Pneumococcal disease§		Legionellosis	Listeriosis	Lyme disease			Malaria
	
		All Ages	Age <5 years			Total	Confirmed	Probable	
**United States**	52	15,635	1,266	3,688	727	30,831	22,014	8,817	1,503
**New England**	1	1,199	77	308	60	11,095	7,455	3,640	104
Connecticut	—	309	17	55	22	2,657	1,653	1,004	21
Maine	1	102	3	18	5	1,111	885	226	5
Massachusetts	—	571	50	173	27	5,138	3,396	1,742	48
New Hampshire	—	80	6	19	3	1,450	1,002	448	9
Rhode Island	—	73	1	31	2	217	133	84	17
Vermont	—	64	—	12	1	522	386	136	4
**Mid. Atlantic**	6	2,290	130	975	166	11,607	8,922	2,685	386
New Jersey	3	596	39	173	44	3,576	2,732	844	67
New York (Upstate)	2	1,045	64	325	43	2,456	1,714	742	42
New York City	—	649	27	177	38	542	330	212	225
Pennsylvania	1	N	N	300	41	5,033	4,146	887	52
**E.N. Central**	7	2,894	228	847	102	2,209	1,765	444	145
Illinois	1	N	49	226	29	204	204	—	43
Indiana	1	728	37	54	10	74	64	10	22
Michigan	3	540	30	178	21	98	80	18	26
Ohio	—	1,149	86	288	28	67	49	18	41
Wisconsin	2	477	26	101	14	1,766	1,368	398	13
**W.N. Central**	2	846	92	171	30	1,735	1,032	703	101
Iowa	—	N	N	13	3	165	92	73	6
Kansas	—	N	N	16	7	19	9	10	7
Minnesota	1	499	31	51	7	1,515	911	604	58
Missouri	1	N	36	68	8	2	1	1	19
Nebraska	—	143	14	11	5	15	5	10	4
North Dakota	—	108	—	3	—	15	10	5	2
South Dakota	—	96	11	9	—	4	4	—	5
**S. Atlantic**	8	3,210	277	613	116	3,842	2,667	1,175	355
Delaware	—	34	2	17	3	669	507	162	2
District of Columbia	—	60	4	N	N	N	N	N	6
Florida	4	988	80	213	33	118	67	51	59
Georgia	—	997	81	56	20	31	31	—	66
Maryland	—	426	31	123	16	1,651	1,113	538	112
North Carolina	2	N	N	65	13	122	27	95	34
South Carolina	1	382	27	26	9	44	35	9	9
Virginia	1	N	36	76	18	1,110	805	305	65
West Virginia	—	323	16	37	4	97	82	15	2
**E.S. Central**	1	1,298	96	137	32	70	24	46	36
Alabama	—	112	16	20	10	25	13	12	10
Kentucky	—	209	11	43	11	14	8	6	10
Mississippi	—	187	25	17	4	1	1	—	4
Tennessee	1	790	44	57	7	30	2	28	12
**W.S. Central**	11	1,967	197	229	41	86	37	49	143
Arkansas	2	185	13	20	6	—	—	—	4
Louisiana	—	247	29	29	2	7	3	4	13
Oklahoma	2	N	26	22	5	4	1	3	24
Texas	7	1,535	129	158	28	75	33	42	102
**Mountain**	6	1,714	142	135	34	44	29	15	75
Arizona	1	661	50	44	14	13	7	6	19
Colorado	—	429	35	24	10	—	—	—	24
Idaho	—	N	1	5	1	5	—	5	8
Montana	—	31	2	4	1	6	6	—	—
Nevada	4	107	9	18	1	10	10	—	8
New Mexico	1	273	20	9	5	1	1	—	2
Utah	—	183	23	27	2	5	2	3	14
Wyoming	—	30	2	4	—	4	3	1	—
**Pacific**	10	217	27	273	146	143	83	60	158
Alaska	—	138	19	1	1	10	4	6	8
California	7	N	N	219	97	70	61	9	108
Hawaii	1	79	8	4	6	N	N	N	4
Oregon	—	N	N	22	16	48	5	43	12
Washington	2	N	N	27	26	15	13	2	26

**Territories**									
American Samoa	—	N	—	N	N	N	N	N	—
C.N.M.I.	—	—	—	—	—	—	—	—	—
Guam	—	—	—	—	—	—	—	—	—
Puerto Rico	—	—	—	2	—	N	N	N	1
U.S. Virgin Islands	—	—	—	—	—	N	N	N	—

Area	Measles			Meningococcal disease				
	
	Total	Indigenous	Imported	All Serogroups	Serogroup A, C, Y, and W-135	Serogroup B	Serogroup Other	Serogroup Unknown
**United States**	55	34	21	551	161	110	20	260
**New England**	1	—	1	15	6	4	2	3
Connecticut	1	—	1	4	2	2	—	—
Maine	—	—	—	3	2	—	1	—
Massachusetts	—	—	—	6	2	1	1	2
New Hampshire	—	—	—	—	—	—	—	—
Rhode Island	—	—	—	—	—	—	—	—
Vermont	—	—	—	2	—	1	—	1
**Mid. Atlantic**	9	1	8	85	17	21	2	45
New Jersey	2	1	1	14	—	—	—	14
New York (Upstate)	1	—	1	21	8	10	—	3
New York City	4	—	4	25	—	—	—	25
Pennsylvania	2	—	2	25	9	11	2	3
**E.N. Central**	17	13	4	72	34	24	6	8
Illinois	—	—	—	17	8	5	4	—
Indiana	15	13	2	8	2	5	—	1
Michigan	1	—	1	13	7	6	—	—
Ohio	1	—	1	25	14	4	1	6
Wisconsin	—	—	—	9	3	4	1	1
**W.N. Central**	6	4	2	40	5	4	—	31
Iowa	—	—	—	2	—	1	—	1
Kansas	6	4	2	6	4	1	—	1
Minnesota	—	—	—	12	1	1	—	10
Missouri	—	—	—	16	—	—	—	16
Nebraska	—	—	—	3	—	—	—	3
North Dakota	—	—	—	1	—	1	—	—
South Dakota	—	—	—	—	—	—	—	—
**S. Atlantic**	4	4	—	83	16	8	2	57
Delaware	1	1	—	1	1	—	—	—
District of Columbia	1	1	—	2	—	—	—	2
Florida	—	—	—	45	—	—	—	45
Georgia	2	2	—	11	5	2	—	4
Maryland	—	—	—	4	3	1	—	—
North Carolina	—	—	—	6	3	2	—	1
South Carolina	—	—	—	5	2	1	2	—
Virginia	—	—	—	5	—	1	—	4
West Virginia	—	—	—	4	2	1	—	1
**E.S. Central**	—	—	—	17	10	3	1	3
Alabama	—	—	—	6	3	—	1	2
Kentucky	—	—	—	1	1	—	—	—
Mississippi	—	—	—	3	—	2	—	1
Tennessee	—	—	—	7	6	1	—	—
**W.S. Central**	4	2	2	58	27	21	2	8
Arkansas	4	2	2	8	5	3	—	—
Louisiana	—	—	—	4	—	—	—	4
Oklahoma	—	—	—	9	3	4	2	—
Texas	—	—	—	37	19	14	—	4
**Mountain**	5	5	—	41	20	6	2	13
Arizona	2	2	—	6	6	—	—	—
Colorado	—	—	—	6	2	4	—	—
Idaho	—	—	—	4	1	—	—	3
Montana	—	—	—	10	4	2	1	3
Nevada	—	—	—	3	1	—	—	2
New Mexico	2	2	—	5	1	—	1	3
Utah	1	1	—	4	2	—	—	2
Wyoming	—	—	—	3	3	—	—	—
**Pacific**	9	5	4	140	26	19	3	92
Alaska	—	—	—	2	—	—	—	2
California	8	5	3	87	—	—	—	87
Hawaii	—	—	—	2	—	—	—	2
Oregon	1	—	1	25	14	10	—	1
Washington	—	—	—	24	12	9	3	—

**Territories**								
American Samoa	—	—	—	—	—	—	—	—
C.N.M.I.	—	—	—	—	—	—	—	—
Guam	—	—	—	—	—	—	—	—
Puerto Rico	2	2	—	—	—	—	—	—
U.S. Virgin Islands	—	—	—	—	—	—	—	—

Area	Mumps	Novel influenza A virus infections†	Pertussis	Plague	Psittacosis	Q fever		

						Total	Acute	Chronic
**United States**	229	313	48,277	4	2	135	113	22
**New England**	8	—	2,594	—	—	1	1	—
Connecticut	—	—	182	—	N	—	—	—
Maine	—	—	737	—	—	—	—	—
Massachusetts	6	—	648	—	—	1	1	—
New Hampshire	—	—	269	—	—	N	N	N
Rhode Island	2	—	113	—	—	—	—	—
Vermont	—	—	645	—	—	N	N	N
**Mid. Atlantic**	30	11	6,511	—	1	10	6	4
New Jersey	—	—	1,395	—	—	3	3	—
New York (Upstate)	6	—	2,715	—	—	3	1	2
New York City	20	—	456	—	—	1	—	1
Pennsylvania	4	11	1,945	—	1	3	2	1
**E.N. Central**	60	275	11,085	—	—	29	29	—
Illinois	32	4	2,026	—	—	4	4	—
Indiana	4	138	441	—	—	2	2	—
Michigan	10	6	845	—	—	3	3	—
Ohio	6	107	893	—	—	2	2	—
Wisconsin	8	20	6,880	—	—	18	18	—
**W.N. Central**	23	10	8,104	—	—	13	8	5
Iowa	6	1	1,736	—	—	N	N	N
Kansas	4	—	887	—	—	2	1	1
Minnesota	7	8	4,142	—	—	—	—	—
Missouri	5	1	815	—	—	3	2	1
Nebraska	1	—	240	—	—	6	3	3
North Dakota	—	—	214	—	—	—	—	—
South Dakota	—	—	70	—	—	2	2	—
**S. Atlantic**	22	15	2,891	—	—	15	15	—
Delaware	—	—	57	—	—	—	—	—
District of Columbia	2	—	26	—	—	N	N	N
Florida	5	—	575	—	—	1	1	—
Georgia	3	—	318	—	—	4	4	—
Maryland	—	12	369	—	—	1	1	—
North Carolina	2	—	612	—	—	9	9	—
South Carolina	1	—	224	—	—	—	—	—
Virginia	7	—	625	—	—	—	—	—
West Virginia	2	3	85	—	—	—	—	—
**E.S. Central**	6	—	1,260	—	—	5	3	2
Alabama	2	—	212	—	—	—	—	—
Kentucky	2	—	666	—	—	3	1	2
Mississippi	—	—	77	—	—	—	—	—
Tennessee	2	—	305	—	—	2	2	—
**W.S. Central**	22	—	2,692	—	—	15	10	5
Arkansas	1	—	248	—	—	1	1	—
Louisiana	2	—	72	—	—	—	—	—
Oklahoma	4	—	154	—	—	2	1	1
Texas	15	—	2,218	—	N	12	8	4
**Mountain**	15	1	6,097	2	—	18	13	5
Arizona	3	—	1,130	—	—	2	1	1
Colorado	7	—	1,494	1	—	9	8	1
Idaho	—	—	235	—	—	1	—	1
Montana	1	—	549	—	—	2	2	—
Nevada	—	—	112	—	—	—	—	—
New Mexico	—	—	924	1	—	1	1	—
Utah	3	1	1,591	—	—	3	1	2
Wyoming	1	—	62	—	—	—	—	—
**Pacific**	43	1	7,043	2	1	29	28	1
Alaska	—	—	353	—	—	—	—	—
California	34	—	795	—	1	22	22	—
Hawaii	1	1	73	—	—	—	—	—
Oregon	6	—	906	2	—	4	4	—
Washington	2	—	4,916	—	—	3	2	1

**Territories**								
American Samoa	—	—	—	—	N	N	N	N
C.N.M.I.	—	—	—	—	—	—	—	—
Guam	4	—	1	—	—	N	N	N
Puerto Rico	4	—	—	—	N	—	—	—
U.S. Virgin Islands	—	—	—	—	—	—	—	—

Area	Rabies		Rubella	Rubella, Congenital syndrome	Salmonellosis	Shiga toxin-producing *E. Coli* (STEC)†

	Animal	Human				
**United States**	4,478	1	9	3	53,800	6,463
**New England**	386	—	1	—	1,993	209
Connecticut	173	—	—	—	444	50
Maine	91	—	—	—	161	20
Massachusetts	—	—	1	—	1,036	96
New Hampshire	28	—	—	—	156	23
Rhode Island	28	—	—	—	108	2
Vermont	66	—	—	—	88	18
**Mid. Atlantic**	832	—	2	—	5,417	675
New Jersey	—	—	—	—	1,147	138
New York (Upstate)	420	—	1	—	1,395	243
New York City	13	—	1	—	1,180	85
Pennsylvania	399	—	—	—	1,695	209
**E.N. Central**	107	—	3	1	5,896	1,176
Illinois	—	—	1	1	1,970	218
Indiana	8	—	1	—	781	181
Michigan	59	—	—	—	995	285
Ohio	40	—	—	—	1,268	238
Wisconsin	N	—	1	—	882	254
**W.N. Central**	252	—	—	—	3,554	1,025
Iowa	33	—	—	—	622	181
Kansas	56	—	—	—	491	97
Minnesota	—	—	—	—	781	258
Missouri	28	—	—	—	1,071	308
Nebraska	—	—	—	—	353	102
North Dakota	75	—	—	—	66	32
South Dakota	60	—	—	—	170	47
**S. Atlantic**	1,334	—	2	1	15,344	610
Delaware	—	—	—	—	148	13
District of Columbia	—	—	—	—	70	8
Florida	103	—	—	—	6,523	93
Georgia	286	—	—	—	2,637	136
Maryland	325	—	2	1	951	74
North Carolina	—	—	—	—	2,200	162
South Carolina	—	—	—	—	1,452	25
Virginia	560	—	—	—	1,144	81
West Virginia	60	—	—	—	219	18
**E.S. Central**	71	—	—	1	4,229	308
Alabama	55	—	—	1	1,150	64
Kentucky	14	—	—	—	732	87
Mississippi	2	—	—	—	1,246	30
Tennessee	—	—	—	—	1,101	127
**W.S. Central**	899	—	—	—	8,697	705
Arkansas	131	—	—	—	1,404	69
Louisiana	4	—	—	—	1,544	27
Oklahoma	81	—	—	—	759	110
Texas	683	—	—	—	4,990	499
**Mountain**	313	—	—	—	2,465	723
Arizona	N	—	—	—	859	141
Colorado	183	—	—	—	509	175
Idaho	23	—	—	—	134	139
Montana	N	—	—	—	109	44
Nevada	18	—	—	—	185	37
New Mexico	47	—	—	—	334	55
Utah	15	—	—	—	260	107
Wyoming	27	—	—	—	75	25
**Pacific**	284	1	1	—	6,205	1,032
Alaska	6	—	—	—	59	N
California	252	1	1	—	4,562	588
Hawaii	—	—	—	—	341	20
Oregon	17	—	—	—	401	192
Washington	9	—	—	—	842	232

**Territories**						
American Samoa	—	—	4	—		
C.N.M.I.	—	—	—	—	—	—
Guam	—	—	—	—	13	—
Puerto Rico	27	—	—	N	165	4
U.S. Virgin Islands	—	—	—	—	—	—

Area	Shigellosis	Spotted Fever Rickettsiosis†			Streptococcal toxic-shock syndrome	Syphilis§		
	
		Total	Confirmed	Probable		All Stages	Congenital (age <2 yr)	Primary & Secondary
**United States**	15,283	4,470	188	4,278	194	49,903	322	15,667
**New England**	212	26	1	25	37	1,118	1	474
Connecticut	46	—	—	—	19	121	—	55
Maine	7	3	—	3	10	22	—	17
Massachusetts	131	7	—	7	2	806	1	316
New Hampshire	8	2	—	2	—	64	—	36
Rhode Island	15	13	—	13	—	93	—	44
Vermont	5	1	1	—	6	12	—	6
**Mid. Atlantic**	2,478	204	6	198	27	7,544	15	1,947
New Jersey	952	128	—	128	10	883	1	229
New York (Upstate)	828	28	5	23	11	939	8	233
New York City	564	7	—	7	—	4,373	—	991
Pennsylvania	134	41	1	40	6	1,349	6	494
**E.N. Central**	2,568	232	11	218	74	5,147	51	1,839
Illinois	280	151	9	142	37	2,423	27	804
Indiana	161	33	2	28	17	531	—	224
Michigan	251	3	—	3	9	786	7	295
Ohio	1,749	23	—	23	10	1,138	16	425
Wisconsin	127	22	—	22	1	269	1	91
**W.N. Central**	973	349	5	344	2	1,111	3	399
Iowa	91	8	—	8	—	143	—	70
Kansas	130	—	—	—	—	129	—	24
Minnesota	390	15	—	15	—	335	1	118
Missouri	71	315	4	311	1	426	1	157
Nebraska	272	9	1	8	1	35	1	8
North Dakota	8	1	—	1	—	14	—	4
South Dakota	11	1	—	1	—	29	—	18
**S. Atlantic**	2,903	1,279	119	1,160	21	11,442	72	3,805
Delaware	22	30	—	30	—	106	1	38
District of Columbia	26	2	1	1	—	589	—	165
Florida	1,702	31	3	28	N	4,483	37	1,369
Georgia	660	92	92	—	—	2,432	14	937
Maryland	222	9	—	9	N	1,243	12	431
North Carolina	136	591	12	579	7	1,036	1	347
South Carolina	37	61	7	54	4	623	6	225
Virginia	91	461	4	457	7	906	1	285
West Virginia	7	2	—	2	3	24	—	8
**E.S. Central**	1,250	950	13	937	8	2,618	7	782
Alabama	332	167	3	164	N	705	4	216
Kentucky	426	62	3	59	8	390	2	150
Mississippi	285	25	2	23	N	456	—	150
Tennessee	207	696	5	691	—	1,067	1	266
**W.S. Central**	2,780	1,332	13	1,319	1	9,560	121	2,222
Arkansas	96	837	5	832	—	468	11	173
Louisiana	215	9	—	9	1	1,779	32	339
Oklahoma	543	409	6	403	N	256	—	83
Texas	1,926	77	2	75	N	7,057	78	1,627
**Mountain**	789	75	11	64	24	2,138	16	698
Arizona	444	50	10	40	—	787	14	202
Colorado	123	6	1	5	2	503	—	208
Idaho	9	4	—	4	—	53	—	26
Montana	11	3	—	3	N	3	—	2
Nevada	55	—	—	—	3	445	1	113
New Mexico	108	4	—	4	—	234	1	101
Utah	34	6	—	6	18	101	—	42
Wyoming	5	2	—	2	1	12	—	4
**Pacific**	1,330	23	9	13	—	9,225	36	3,501
Alaska	7	N	—	—	—	34	1	11
California	1,071	21	8	12	N	8,015	34	2,953
Hawaii	27	N	N	N	—	43	—	23
Oregon	92	1	—	1	N	424	1	212
Washington	133	1	1	—	N	709	—	302

**Territories**								
American Samoa	5	N	N	N	N	—	—	—
C.N.M.I.	—	—	—	—	—	—	—	—
Guam	1	N	N	N	—	27	—	6
Puerto Rico	2	N	N	N	N	704	1	306
U.S. Virgin Islands	—	N	N	N	—	2	—	—

Area	Tetanus	Toxic-shock syndrome	Trichinellosis	Tuberculosis†	Tularemia
**United States**	37	65	18	9,945	149
**New England**	—	—	—	342	8
Connecticut	—	N	—	74	—
Maine	—	—	—	17	—
Massachusetts	—	—	—	215	8
New Hampshire	—	—	—	9	—
Rhode Island	—	—	—	23	—
Vermont	—	—	—	4	—
**Mid. Atlantic**	4	18	2	1,402	—
New Jersey	—	3	2	302	—
New York (Upstate)	—	9	—	215	—
New York City	1	—	—	651	—
Pennsylvania	3	6	—	234	—
**E.N. Central**	8	12	4	818	8
Illinois	1	4	1	347	4
Indiana	3	1	—	102	4
Michigan	2	6	1	149	—
Ohio	2	1	—	149	—
Wisconsin	—	—	2	71	—
**W.N. Central**	3	9	1	406	64
Iowa	—	1	—	46	1
Kansas	—	—	—	42	22
Minnesota	2	4	1	162	—
Missouri	1	4	—	89	27
Nebraska	—	—	—	22	6
North Dakota	—	—	—	26	3
South Dakota	—	—	—	19	5
**S. Atlantic**	7	11	4	1,901	5
Delaware	—	1	—	28	—
District of Columbia	—	—	1	37	—
Florida	4	N	—	679	—
Georgia	—	9	N	357	—
Maryland	—	N	1	224	2
North Carolina	—	—	—	211	1
South Carolina	2	1	—	122	—
Virginia	1	N	2	235	2
West Virginia	—	—	—	8	—
**E.S. Central**	2	3	—	459	6
Alabama	1	—	—	134	—
Kentucky	—	—	N	80	4
Mississippi	1	N	—	81	—
Tennessee	—	3	—	164	2
**W.S. Central**	5	1	1	1,540	39
Arkansas	—	1	N	70	22
Louisiana	—	—	—	149	—
Oklahoma	2	N	—	88	17
Texas	3	N	1	1,233	—
**Mountain**	2	4	1	457	10
Arizona	—	1	1	211	—
Colorado	1	—	—	64	1
Idaho	—	1	—	15	1
Montana	—	N	—	5	3
Nevada	—	—	—	82	1
New Mexico	1	—	—	40	1
Utah	—	2	—	37	2
Wyoming	—	—	—	3	1
**Pacific**	6	7	5	2,620	9
Alaska	1	N	5	66	2
California	4	7	—	2,191	2
Hawaii	—	N	—	117	—
Oregon	—	N	—	61	—
Washington	1	N	—	185	5

**Territories**					
American Samoa	—	N	N	1	—
C.N.M.I.	—	—	—	21	—
Guam	—	—	—	68	—
Puerto Rico	1	N	N	71	—
U.S. Virgin Islands	—	—	—	4	—

Area	Typhoid fever	Vancomycin-intermediate *Staphylococcus aureus*	Vancomycin-resistant *Staphylococcus aureus*	Varicella		Vibriosis§

				Morbidity	Mortality†	
**United States**	354	134	2	13,447	3	1,111
**New England**	17	3	—	1,424	—	118
Connecticut	2	—	—	265	—	24
Maine	—	—	—	258	—	10
Massachusetts	12	2	—	534	N	70
New Hampshire	1	N	—	142	—	3
Rhode Island	2	1	—	80	—	11
Vermont	—	—	—	145	N	—
**Mid. Atlantic**	97	40	—	1,327	1	80
New Jersey	20	3	—	466	1	41
New York (Upstate)	22	30	—	N	N	N
New York City	45	4	—	—	—	27
Pennsylvania	10	3	—	861	—	12
**E.N. Central**	47	25	—	3,583	—	51
Illinois	14	4	—	898	—	22
Indiana	4	N	—	469	—	6
Michigan	9	10	—	971	—	7
Ohio	15	9	—	806	N	11
Wisconsin	5	2	—	439	—	5
**W.N. Central**	13	21	—	881	—	27
Iowa	3	N	—	N	N	N
Kansas	1	N	N	395	—	N
Minnesota	5	1	—	—	—	15
Missouri	3	20	—	388	—	8
Nebraska	—	—	—	27	—	2
North Dakota	1	—	—	39	—	2
South Dakota	—	—	—	32	N	N
**S. Atlantic**	46	16	1	1,611	—	322
Delaware	—	—	1	3	—	6
District of Columbia	1	2	—	19	—	4
Florida	11	7	—	816	—	147
Georgia	12	1	—	51	—	29
Maryland	7	1	—	N	—	53
North Carolina	4	2	—	N	N	31
South Carolina	1	—	—	11	—	11
Virginia	10	2	—	505	N	41
West Virginia	—	1	—	206	—	N
**E.S. Central**	5	1	1	201	—	55
Alabama	1	1	—	190	N	20
Kentucky	—	N	N	N	N	2
Mississippi	1	—	—	11	N	16
Tennessee	3	—	1	N	—	17
**W.S. Central**	33	26	—	2,715	1	119
Arkansas	2	—	—	236	—	N
Louisiana	2	1	—	69	N	53
Oklahoma	—	2	—	N	N	—
Texas	29	23	—	2,410	1	66
**Mountain**	17	2	—	1,578	—	45
Arizona	7	2	—	535	—	29
Colorado	7	N	—	483	N	10
Idaho	—	N	N	N	N	N
Montana	—	N	N	132	—	N
Nevada	1	—	—	N	N	4
New Mexico	—	N	N	99	—	1
Utah	2	—	—	310	—	1
Wyoming	—	—	—	19	N	—
**Pacific**	79	—	—	127	1	294
Alaska	—	N	N	58	N	3
California	61	N	N	24	1	170
Hawaii	5	—	—	45	—	35
Oregon	2	N	N	N	N	19
Washington	11	N	—	N	—	67

**Territories**						
American Samoa	4	N	N	N	N	N
C.N.M.I.	—	—	—	—	—	—
Guam	—	—	—	50	N	1
Puerto Rico	—	—	—	199	—	N
U.S. Virgin Islands	—	—	—	—	—	—

N: Not Reportable U: Unavailable —: No reported cases C.N.M.I.: Commonwealth of the Northern Mariana Islands.

* No cases of anthrax; eastern equine encephalitis virus disease, nonneuroinvasive; poliomyelitis, paralytic; poliovirus infection, nonparalytic; Powassan virus nonneuroinvasive disease; severe acute respiratory syndrome-associated coronavirus disease (SARS-CoV); smallpox; western equine encephalitis virus disease, neuroinvasive and non-neuroinvasive; yellow fever; and viral hemorrhagic fevers were reported in the United States during 2012. Data on chronic hepatitis B and hepatitis C virus infection (past or present) are not included because they are undergoing data quality review.

† Totals reported to the Division of Vector-Borne Infectious Diseases (DVBD), National Center for Emerging and Zoonotic Infectious Diseases (NCZVED) (ArboNET Surveillance), as of June 1, 2013.

N: Not Reportable U: Unavailable —: No reported cases C.N.M.I.: Commonwealth of the Northern Mariana Islands.

* No cases of anthrax; eastern equine encephalitis virus disease, nonneuroinvasive; poliomyelitis, paralytic; poliovirus infection, nonparalytic; Powassan virus nonneuroinvasive disease; severe acute respiratory syndrome-associated coronavirus disease (SARS-CoV); smallpox; western equine encephalitis virus disease, neuroinvasive and non-neuroinvasive; yellow fever; and viral hemorrhagic fevers were reported in the United States during 2012. Data on chronic hepatitis B and hepatitis C virus infection (past or present) are not included because they are undergoing data quality review.

† Includes cases reported as wound and unspecified botulism.

§ Totals reported to the Division of STD Prevention, NCHHSTP, as of May 29, 2013.

N: Not Reportable U: Unavailable —: No reported cases C.N.M.I.: Commonwealth of the Northern Mariana Islands.

* No cases of anthrax; eastern equine encephalitis virus disease, nonneuroinvasive; poliomyelitis, paralytic; poliovirus infection, nonparalytic; Powassan virus nonneuroinvasive disease; severe acute respiratory syndrome-associated coronavirus disease (SARS-CoV); smallpox; western equine encephalitis virus disease, neuroinvasive and non-neuroinvasive; yellow fever; and viral hemorrhagic fevers were reported in the United States during 2012. Data on chronic hepatitis B and hepatitis C virus infection (past or present) are not included because they are undergoing data quality review.

† Totals reported to the Division of STD Prevention, NCHHSTP, as of May 29, 2013.

N: Not Reportable U: Unavailable —: No reported cases C.N.M.I.: Commonwealth of the Northern Mariana Islands.

* No cases of anthrax; eastern equine encephalitis virus disease, nonneuroinvasive; poliomyelitis, paralytic; poliovirus infection, nonparalytic; Powassan virus nonneuroinvasive disease; severe acute respiratory syndrome-associated coronavirus disease (SARS-CoV); smallpox; western equine encephalitis virus disease, neuroinvasive and non-neuroinvasive; yellow fever; and viral hemorrhagic fevers were reported in the United States during 2012. Data on chronic hepatitis B and hepatitis C virus infection (past or present) are not included because they are undergoing data quality review.

† Total number of reported laboratory-positive dengue cases including all confirmed cases [by anti-dengue virus (DENV) molecular diagnostic methods or seroconversion of anti-DENV IgM] and all probable cases (by a single, positive anti-DENV IgM). Totals reported to the DVBD, NCEZID (ArboNET Surveillance), as of June 1, 2013.

N: Not Reportable U: Unavailable —: No reported cases C.N.M.I.: Commonwealth of the Northern Mariana Islands.

* No cases of anthrax; eastern equine encephalitis virus disease, nonneuroinvasive; poliomyelitis, paralytic; poliovirus infection, nonparalytic; Powassan virus nonneuroinvasive disease; severe acute respiratory syndrome-associated coronavirus disease (SARS-CoV); smallpox; western equine encephalitis virus disease, neuroinvasive and non-neuroinvasive; yellow fever; and viral hemorrhagic fevers were reported in the United States during 2012. Data on chronic hepatitis B and hepatitis C virus infection (past or present) are not included because they are undergoing data quality review.

† Totals reported to the Division of STD Prevention, NCHHSTP, as of May 29, 2013.

N: Not Reportable U: Unavailable —: No reported cases C.N.M.I.: Commonwealth of the Northern Mariana Islands.

* No cases of anthrax; eastern equine encephalitis virus disease, nonneuroinvasive; poliomyelitis, paralytic; poliovirus infection, nonparalytic; Powassan virus nonneuroinvasive disease; severe acute respiratory syndrome-associated coronavirus disease (SARS-CoV); smallpox; western equine encephalitis virus disease, neuroinvasive and non-neuroinvasive; yellow fever; and viral hemorrhagic fevers were reported in the United States during 2012. Data on chronic hepatitis B and hepatitis C virus infection (past or present) are not included because they are undergoing data quality review.

† Total number of HIV cases reported to the Division of HIV/AIDS Prevention, National Center for HIV/AIDS, Viral Hepatitis, STD, and TB Prevention (NCHHSTP) through December 31, 2012.

N: Not Reportable U: Unavailable —: No reported cases C.N.M.I.: Commonwealth of the Northern Mariana Islands.

* No cases of anthrax; eastern equine encephalitis virus disease, nonneuroinvasive; poliomyelitis, paralytic; poliovirus infection, nonparalytic; Powassan virus nonneuroinvasive disease; severe acute respiratory syndrome-associated coronavirus disease (SARS-CoV); smallpox; western equine encephalitis virus disease, neuroinvasive and non-neuroinvasive; yellow fever; and viral hemorrhagic fevers were reported in the United States during 2012. Data on chronic hepatitis B and hepatitis C virus infection (past or present) are not included because they are undergoing data quality review.

† Totals reported to the Influenza Division, National Center for Immunization and Respiratory Diseases (NCIRD), as of December 31, 2012.

§ *Streptococcus pneumoniae*, invasive disease. The previous categories of invasive pneumococcal disease among children less than 5 years and invasive, drug-resistant Streptococcus pneumoniae were eliminated. All cases of invasive Streptococcus pneumoniae disease, regardless of age or drug resistance are reported under a single disease code.

N: Not Reportable U: Unavailable —: No reported cases C.N.M.I.: Commonwealth of the Northern Mariana Islands.

* No cases of anthrax; eastern equine encephalitis virus disease, nonneuroinvasive; poliomyelitis, paralytic; poliovirus infection, nonparalytic; Powassan virus nonneuroinvasive disease; severe acute respiratory syndrome-associated coronavirus disease (SARS-CoV); smallpox; western equine encephalitis virus disease, neuroinvasive and non-neuroinvasive; yellow fever; and viral hemorrhagic fevers were reported in the United States during 2012. Data on chronic hepatitis B and hepatitis C virus infection (past or present) are not included because they are undergoing data quality review.

N: Not Reportable U: Unavailable —: No reported cases C.N.M.I.: Commonwealth of the Northern Mariana Islands.

* No cases of anthrax; eastern equine encephalitis virus disease, nonneuroinvasive; poliomyelitis, paralytic; poliovirus infection, nonparalytic; Powassan virus nonneuroinvasive disease; severe acute respiratory syndrome-associated coronavirus disease (SARS-CoV); smallpox; western equine encephalitis virus disease, neuroinvasive and non-neuroinvasive; yellow fever; and viral hemorrhagic fevers were reported in the United States during 2012. Data on chronic hepatitis B and hepatitis C virus infection (past or present) are not included because they are undergoing data quality review.

† Totals reported to the Influenza Division, NCIRD, as of December 31, 2012.

N: Not Reportable U: Unavailable —: No reported cases C.N.M.I.: Commonwealth of the Northern Mariana Islands.

* No cases of anthrax; eastern equine encephalitis virus disease, nonneuroinvasive; poliomyelitis, paralytic; poliovirus infection, nonparalytic; Powassan virus nonneuroinvasive disease; severe acute respiratory syndrome-associated coronavirus disease (SARS-CoV); smallpox; western equine encephalitis virus disease, neuroinvasive and non-neuroinvasive; yellow fever; and viral hemorrhagic fevers were reported in the United States during 2012. Data on chronic hepatitis B and hepatitis C virus infection (past or present) are not included because they are undergoing data quality review.

† Includes *E. coli* O157:H7; Shiga toxin-positive, serogroup non-O157; and Shiga toxin positive, not serogrouped.

N: Not Reportable U: Unavailable —: No reported cases C.N.M.I.: Commonwealth of the Northern Mariana Islands.

* No cases of anthrax; eastern equine encephalitis virus disease, nonneuroinvasive; poliomyelitis, paralytic; poliovirus infection, nonparalytic; Powassan virus nonneuroinvasive disease; severe acute respiratory syndrome-associated coronavirus disease (SARS-CoV); smallpox; western equine encephalitis virus disease, neuroinvasive and non-neuroinvasive; yellow fever; and viral hemorrhagic fevers were reported in the United States during 2012. Data on chronic hepatitis B and hepatitis C virus infection (past or present) are not included because they are undergoing data quality review.

† Total case count includes four unknown case status reports.

§ Includes the following categories: primary, secondary, latent (including early latent, late latent, and latent syphilis of unknown duration), neurosyphilis, late (including late syphilis with clinical manifestations other than neurosyphilis), and congenital syphilis. Totals reported to the Division of STD Prevention, NCHHSTP, as of May 29, 2013.

N: Not Reportable U: Unavailable —: No reported cases C.N.M.I.: Commonwealth of the Northern Mariana Islands.

* No cases of anthrax; eastern equine encephalitis virus disease, nonneuroinvasive; poliomyelitis, paralytic; poliovirus infection, nonparalytic; Powassan virus nonneuroinvasive disease; severe acute respiratory syndrome-associated coronavirus disease (SARS-CoV); smallpox; western equine encephalitis virus disease, neuroinvasive and non-neuroinvasive; yellow fever; and viral hemorrhagic fevers were reported in the United States during 2012. Data on chronic hepatitis B and hepatitis C virus infection (past or present) are not included because they are undergoing data quality review.

† Totals reported to the Division of Tuberculosis Elimination, NCHHSTP, as of June 15, 2013.

N: Not Reportable U: Unavailable —: No reported cases C.N.M.I.: Commonwealth of the Northern Mariana Islands.

* No cases of anthrax; eastern equine encephalitis virus disease, nonneuroinvasive; poliomyelitis, paralytic; poliovirus infection, nonparalytic; Powassan virus nonneuroinvasive disease; severe acute respiratory syndrome-associated coronavirus disease (SARS-CoV); smallpox; western equine encephalitis virus disease, neuroinvasive and non-neuroinvasive; yellow fever; and viral hemorrhagic fevers were reported in the United States during 2012. Data on chronic hepatitis B and hepatitis C virus infection (past or present) are not included because they are undergoing data quality review.

† Totals reported to the Division of Viral Diseases, NCIRD, as of May 1, 2013.

§ Vibriosis refers to any species of the family Vibrionaceae, other than toxigenic Vibrio cholerae O1 or O139.
