# Erratum

**Published:** 2013-08-30

## Vol. 62, No. 33

In the Notice to Readers, “Final 2012 Reports of Nationally Notifiable Infectious Diseases,” on page 669, the first sentence should read as follows: “The tables listed in this report on pages **670–682** summarize finalized data, as of June 30, 2013, from the National Notifiable Diseases Surveillance System (NNDSS) for 2012.

